# Constitutional *BRCA1* Epimutations: A Key for Understanding Basal-Like Breast and High-Grade Serous Ovarian Cancer

**DOI:** 10.1155/2024/7353984

**Published:** 2024-09-15

**Authors:** Per E. Lønning, Oleksii Nikolaienko, Stian Knappskog

**Affiliations:** ^1^ K.G. Jebsen Center for Genome-Directed Cancer Therapy Department of Oncology Haukeland University Hospital, Bergen, Norway; ^2^ Department of Clinical Science University of Bergen, Bergen, Norway

**Keywords:** *BRCA1*, cancer risk, constitutional, epimutations, hypermethylation, ovarian cancer, triple-negative breast cancer

## Abstract

Germline pathogenic genetic variants in the *BRCA1* and *BRCA2* genes are the most frequent causes of familial breast and ovarian cancer. Contrasting *BRCA2,* epimutations in the *BRCA1* gene are frequently detected in tissue from triple-negative breast (TNBC) and high-grade serous ovarian cancers (HGSOC). While studies over the last decade have reported *BRCA1* epimutations in white blood cells (WBC) from breast and ovarian cancer patients, the potential hazard ratio for incident TNBC and HGSOC was not formally assessed until recently.

Conducting a prospective nested case-control study on women participating in the American Women's Health Initiative Study, we provided firm evidence that mosaic WBC *BRCA1* epimutations, even at allele frequencies < 0.1%, are associated with a significantly increased risk of both incident HGSOC and TNBC > 5 years after WBC collection. In a second study assessing *BRCA1* epimutations in WBC and matched tumor samples from TNBC, our results indicated such epimutations to be the underlying cause of around 20% of TNBC, far exceeding the percentage of cases carrying *BRCA1* germline pathogenic genetic variants.

We detected primary constitutional *BRCA1* epimutations in tissues derived from all three germ layers. They occur independently of *BRCA1* promoter haplotypes but are present on the same allele in all WBC within affected individuals. Moreover, epimutations are consistently found on the same allele in normal and tumor breast tissue as well as in WBC. This finding, together with *BRCA1* epimutations detected in WBC from newborns, strongly indicates an early embryonic event with clonal expansion affecting all germ layers.

Future work in the field must lead to an understanding of exactly when and how the *BRCA1* epimutations occur and, most importantly, whether primary constitutional epimutations in genes other than *BRCA1* may cause an elevated risk of other cancer types.

## 1. Introduction

Women harboring germline pathogenic variants (PV) in the breast cancer Type 1 susceptibility gene (*BRCA1)* have a lifetime risk of breast and high-grade serous ovarian cancer (HGSOC) exceeding 70% and 40%, respectively [1]. For breast cancer, the risk is particularly high for the so-called triple-negative subtype (TNBC) [2]. While the fraction of breast cancers that are TNBC varies between 10% and 20% among ethnic groups [3], about 70% of breast cancer cases arising in women harboring a PV in *BRCA1* are TNBCs [4].

About 80% of all TNBCs present a basal-like gene expression signature [5–7]. Further, 40%–70% present signatures of homologous recombination repair deficiency (HRD) and RAD51 foci deficiency [8–12], similar to what is seen with *BRCA1* or *BRCA2* deficiency. However, only between 8% and 40% of all TNBCs (number pending on ethnic group) carry a germline *BRCA1* PV and very few harbor *BRCA2* deficits as an underlying cause of their disease [13–15]. As for HGSOC, approximately 50% reveal a gene expression signature of HRD [16], while the incidence of *BRCA1* and *BRCA2* PV range between 8%–15% and 4%–8%, respectively [17–19]. While studies have identified germline PV in other genes involved in homologous recombination repair (HRR) like *PALB2*, *BRIP1*, *RAD51C*, and *RAD51D* in TNBC and HGSOC, such mutations, similar to *somatic* mutations in *BRCA1*, and *BRCA2*, are rare events [2, 15, 17, 20–22]. Moreover, the fact that TNBC and HGSOC have been meticulously characterized by whole genome sequencing (WGS) over the last decade leaves the likelihood of identifying new, previously unknown, aberrations relatively low. Taken together, a high fraction of TNBC and HGSOC reveal BRCA-like molecular features without having a PV either in *BRCA1/2* or other genes involved in the same pathways.

The lack of new genetic findings associated with high TNBC penetrance argues for alternative explanations. Epigenetic (de)regulation is one such potential explanation for the pathology and etiology of many cases of TNBC and HGSOC.

## 2. *BRCA1* Epimutations in TNBC and HGSOC Tissue

While the terms “epigenetics” and “epimutations” cover a number of biological processes including both histone modifications and DNA methylation [23], gene promoter hypermethylation and histone modifications hindering transcription often appear in concert [24]. Further, since promoter hypermethylation is the epigenetic mechanism most often associated with cancer biology in general and *BRCA1* transcriptional activity in particular [25–27], here, we will use the term *BRCA1* epimutation synonymously to *BRCA1* promoter hypermethylation.

**Figure 1:**
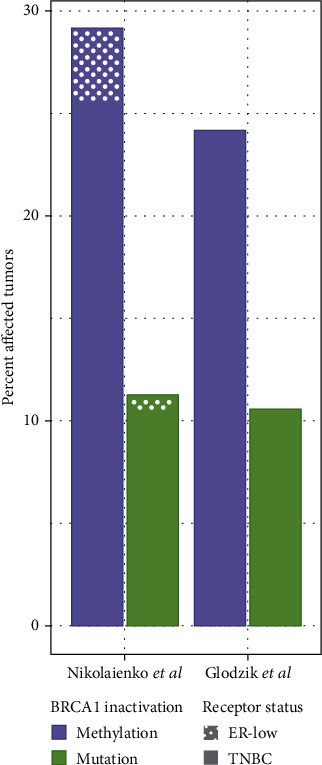
Fraction of *BRCA1*-methylated (constitutional and somatic; purple bars) versus *BRCA1-*mutated (germline or somatic; green bars) TNBCs in two recent studies conducted on Scandinavian populations [26, 31]. In the study by Nikolaienko et al. [31], patients harboring ER low-level tumors (1%–10%) are included together with the TNBC (dotted area).

Recent studies have shown 25%–30% of primary TNBC and 9%–20% of HGSOC to harbor *BRCA1* epimutations [26, 28–33]. Thus, for TNBC, the incidence of *BRCA1* tumor epimutations is around two-fold higher than the combined incidence of somatic (*sBRCA1*) and germline (g*BRCA1*) mutations (Figure 1) [21, 26, 31]. Like *BRCA1* mutations, *BRCA1* epimutations are known to act according to the classical Knudson hypothesis requiring the inactivation of both alleles [15, 21], most often by the epimutation event being followed by LOH [26, 34]. Analyzing large sets of TNBC with the use of WGS and RNA sequencing, no difference in genomic patterns, including mutational signatures, between tumors harboring germline or somatic *BRCA1* PV and those harboring *BRCA1* epimutations has been recorded [10, 15, 21, 26]. This finding argues for *BRCA1* epimutations as well as somatic *BRCA1* mutations to be early events during tumor evolution.

Noteworthy, when assessing *BRCA1* epimutations in non-TNBC breast cancers [31], *BRCA1* hypermethylation was frequently observed in the small group of so-called ER-low tumors (tumors revealing ER immunostaining between 1% and 10%), which has been shown to have gene expression signatures mirroring TNBC [35]. As for the rest of the non-TNBC, the so-called HER2-enriched and the luminal cancers having an ER expression > 10%, *BRCA1* epimutations were observed in about 2% only. Thus, in these non-TNBC cases, PV in other genes associated with HRR were more frequent than the *BRCA1* epimutations [36]. Similarly, in ovarian cancer, *BRCA1* epimutations are infrequently recorded in low-grade serous as well as nonserous tumors as compared to more frequent observations in HGSOC [37].

A potential difference between epimutations and *BRCA1* PV relates to their influence on acquired drug resistance [38]. *BRCA1* promoter hypermethylation has been shown to cause transcriptional repression [25, 27], and both epimutations and *BRCA1* PV have been recorded as markers for sensitivity to PARP inhibition and chemotherapy for TNBC in the primary setting [26, 39, 40]. However, in the large TNT study on metastatic TNBC, only *gBRCA1* PV, not *BRCA1* epimutations, were associated with improved response to platinum therapy [7]. While loss of *BRCA1* promoter methylation during therapy has been described in ovarian cancer [41], notably, such loss of methylation may not explain therapy resistance in the TNT trial since methylation status was determined in samples collected at trial inclusion [7]. Clearly, more research is needed to characterize interactions between *BRCA1* epimutation status and therapy sensitivity in breast as well as ovarian cancer.

## 3. Constitutional Epimutations in *BRCA1* and Cancer Risk

**Figure 2:**
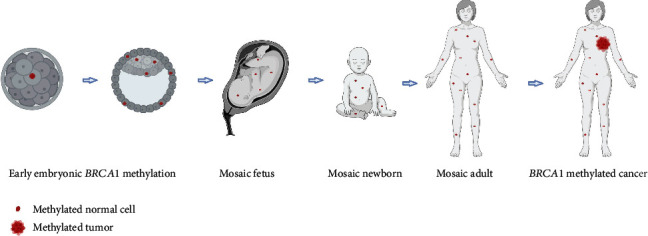
General model depicting early prenatal (constitutional) *BRCA1* hypermethylation as an underlying contributor to TNBC. Red dots represent *BRCA1*-epimutated normal cells, appearing through an early embryonic event, resulting in a mosaic adult. Red cell group represents breast cancer. Reproduced with permission from [31].

Constitutional epimutations refer to normal tissue epimutations occurring prenatally, generally affecting all three germline layers (Figure 2). These epimutations can be classified into two major groups: primary constitutional epimutations, not associated with any genetic aberrations, and secondary epimutations, associated with a genetic aberration [42, 43].

In 2018, Evans et al. [44] reported a dominantly inherited 5′ UTR variant associated with epigenetic *BRCA1* silencing due to promoter hypermethylation in two families affected with a high incidence of breast and ovarian cancer. To this end, no additional families harboring either this or other promoter variants associated with secondary *BRCA1* promoter hypermethylation have been reported, indicating secondary *BRCA1* epimutations to be a rare event.

Following the first report on mosaic *BRCA1* epimutations in white blood cells (WBC) from breast cancer patients in 2008 [45], a number of studies confirmed the same observation among breast and ovarian cancer patients [45–51]. These studies, however, enrolled a limited number of patients, preventing risk calculations. In 2018, using a large hospital-based cohort of ovarian cancer patients (*n* = 934) and population-based controls (*n* = 1698), followed by a similar verification cohort (*n* = 607 patients and *n* = 1984 controls), we found low-level mosaic *BRCA1* epimutations to be associated with an odds ratio of 2.2–2.9 for HGSOC, but no increased risk for other types of ovarian cancer [52]. This study, however, like all previous studies, was conducted on WBC samples drawn after the patients had been diagnosed with their cancers and there may be tumor-derived DNA in the circulation. The risk of tumor DNA contamination in DNA extracted from the WBC fraction, however, is low, taking into account the fraction of circulating tumor cells versus WBC detected in the circulation estimated to be less than 1 to a million. Moreover, the concentration of free tumor DNA in the plasma is far lower than the DNA derived from WBC [53–55]. A second issue is potential alterations in WBC fractions. Studies applying genome-wide methylation analyses have detected differences in WBC DNA methylation related to incident cancers likely due to changes in WBC fractions [56–67]. Variations between WBC fractions also need to be taken into consideration when comparing WBC epimutations between newborns and adults. As for *BRCA1* epimutations, examining publicly available datasets [59, 64], we detected no variation in *BRCA1* promoter methylation patterns with respect to WBC subfractions, neither in newborns nor adults [52]. Taken together, while these findings strongly indicate that the risk of tumor-directed influence on WBC *BRCA1* methylation in patients diagnosed with cancer is low, in theory, such secondary effects may not be excluded.

In a subsequent population-based nested case-control study in the Women's Health Initiative (WHI), we found mosaic *BRCA1* epimutations in WBC of healthy women to be associated with an elevated hazard ratio (HR) for incident HGSOC of 1.93 and TNBC of 2.35 [68]. Importantly, similar HRs were found in the subgroup analysis of patients and controls from whom WBC samples were collected > 5 years prior to their cancer diagnosis. The findings represent proof-of-concept for constitutional *BRCA1* epimutations being a cancer risk factor.

**Figure 3:**
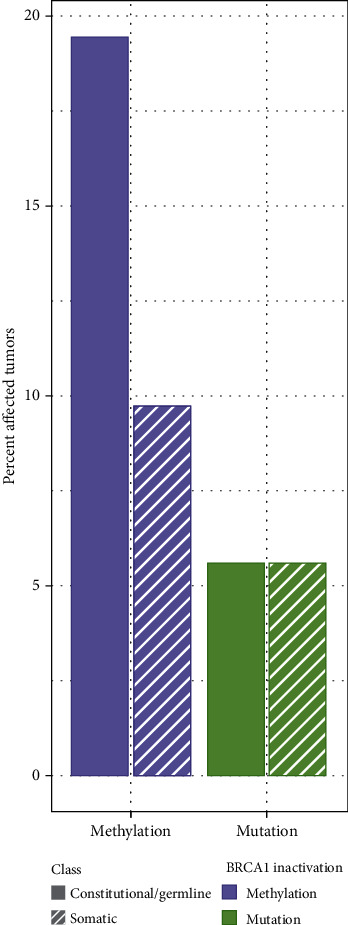
Fractions of TNBC and HER2–/ER < 10% tumors (*n* = 72) characterized by different molecular mechanisms of *BRCA1* inactivation. Bars indicate the fraction of tumors with methylation (purple) or mutation (green) split on their potential time of emergence, as constitutional/germline (solid fill) or somatic (striped pattern). Reproduced with permission from [31].

Mosaic *BRCA1* epimutations are observed at similar variant allele frequency (VEF) when detected in healthy women subsequently diagnosed with an incident TNBC or HGSOC many years later or detected in cancer patients after diagnosis. This indicates that WBC methylation should be interpreted as constitutional epimutations and not secondary cancer-related effects. This further justifies *BRCA1* epimutation analysis in studies making use of normal tissue samples collected after diagnosis. Some questions are optimally addressed by studying tumor and WBC samples collected at the same time in the patient [31]. In addition, this finding opens a possibility for reassessing previous results. The somewhat lower HR for HGSOC (1.93) in the WHI study [68] as compared to our previous results (HR: 2.22–2.91) [52] questions whether the HRs in the WHI study may represent underestimates. *BRCA1-*hypermethylated breast cancers on average are diagnosed at a younger age compared to sporadic TNBC [26]. With a median age of 63 years at enrolment for our participants in the WHI study [68], it is likely that a number of TNBC as well as HGSOC may have been detected prior to study inclusion. This aligns with the findings by others in TNBC: Prajzendanc et al. reported an OR of 4.7 for TNBC comparing hospital-based patients and healthy controls [69]. Finally, recent findings by us [31] assessing tumor and WBC *BRCA1* methylation in concert suggested about 20% of all TNBC and ER low-expression tumors may arise from cells harboring constitutional *BRCA1* hypermethylation (Figure 3). Notably, this percentage is well above the incidence of TNBC harboring genetic variants (somatic and germline taken together) as detected in the same study as well as in a similar ethnic population [26].

## 4. Characteristics of Primary Constitutional *BRCA1* Epimutations

**Figure 4:**
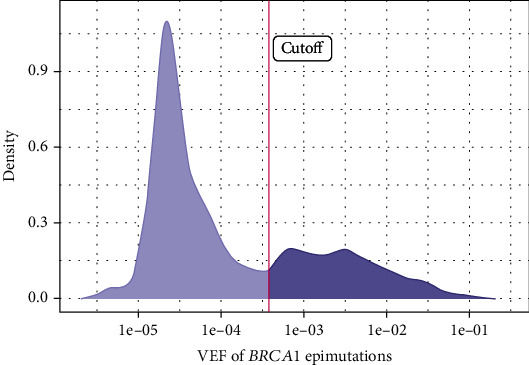
Distribution of intraindividual epiallele frequencies (VEF) of *BRCA1* epimutations [70]. Density is based on combined values from all samples (cases and controls) analyzed in our Women's Health Initiative study. Vertical red line represents the cutoff value for methylation positivity. Reproduced with permission from supporting information in reference in [68].

The observed incidence of low-level mosaic epimutations depends on the sensitivity of the applied assay and the set cutoff. In our study, employing a highly sensitive NGS-based assay [70], WBC *BRCA1* epimutations with a VEF as low as 0.04% (Figure 4) were detected in 5.5% of healthy (noncancer controls) US women (WHI; median age 63 years; range 50–79 years) [68]. While a VEF of 17% has been recorded, the majority reveal a VEF of < 2% (see supporting information in [68] and Figure 4). Moreover, we were able to show a methylation profile with near to full methylation of all CpGs in the *BRCA1* promoter region, on a limited number of alleles. Thus, methylation status may be quantitated based on VEF. Notably, the result with respect to cancer risk aligned well between VEF and conventional *β*-value-based analyses [68].

The presence of heavily methylated alleles indirectly justifies the use of less costly but sensitive methods determining *BRCA1* epimutations. In our previous study on HGSOC, we applied a qPCR-based assay [52], and Lubinski's group in Poland developed a MS-HRM assay [69, 71] for WBC *BRCA1* methylation analysis. Both these assays detect low-VEF *BRCA1* epimutations and could potentially be used for large-scale screens, although a head-to-head comparison of the sensitivity of the different assays has not been performed. Notably, both groups [70, 71] found the EPIC arrays to lack the sensitivity to detect the majority of WBC *BRCA1* epimutations. It should be emphasized that this relates to *BRCA1* primary epimutations. Regarding potential primary epimutations affecting other genes, methylation status across individual alleles would need to be confirmed using next-generation sequencing or similar allele-specific, single CpG-resolution techniques, like pyrosequencing, prior to applying simpler methods for screening.

Notably, it may be of importance to detect the accurate level of methylation (e.g., VEF) and not merely score samples as positive or negative. In our first study on ovarian cancer [52], we observed a nonsignificant trend for a higher HR of HGSOC related to a VEF above as compared to below median level, but this was not confirmed in our subsequent WHI study [68].

**Figure 5:**
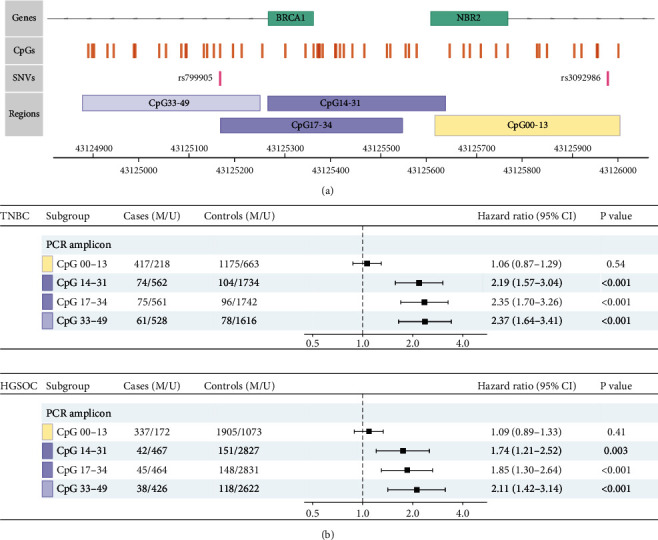
(a) The genomic structure of the *BRCA1* bidirectional promoter region. The first exons of the *BRCA1* and *NBR2* genes are depicted by green rectangles, covered CpGs are indicated by orange vertical lines, rs799905 single-nucleotide variation by pink vertical line, and the four sequenced amplicons by blue rectangles. (b) Risk of incident TNBC or HGSOC according to methylation called in four PCR amplicons (assessed by VEF) covering different but partly overlapping regions of the *BRCA1* promoter. Reproduced with permission from [68].

The *BRCA1* gene promoter is bidirectional; in addition to *BRCA1,* it regulates the expression of the non-protein coding Next-To-*BRCA1* type-2 (*NBR2*) gene [72]. To assess regional characteristics of *BRCA1* promoter epimutations with respect to TNBC and HGSOC risk, we analyzed CpG methylation across the core promoter area as well as within the *NBR2* and *BRCA1* gene bodies (Figure 5). Contrasting methylation in the core promoter and the *BRCA1* upstream region, CpG methylation within the *NBR2* gene body had no impact on either TNBC or HGSOC HR [68].

To confirm *BRCA1* epimutations to be constitutional, we determined *BRCA1* methylation status in umbilical cord blood. Doing so, we detected *BRCA1* epimutations in about 9% of newborn females, contrasting an incidence of about 4.5% in newborn males. Examining methylation status in a subset of parents, epimutation frequency was about 8% in young women, contrasting 3% in young adult males [31]. Most importantly, no correlation between newborn and parental epimutation status was recorded, arguing against Mendelian inheritance. Moreover, while *BRCA1* epimutations occurred independent of *BRCA1* promoter genotype assessed by rs799905 SNP status [68], within each affected individual, the same allele carried the epimutation across tissues. The latter concordance in allele specificity was also observed between WBC, normal breast, and TNBC tissue collected from the same patients [31].

The frequency of methylation across different groups merits considerations. Currently, we lack an explanation to the significant difference in methylation frequency between males and females. However, within the group of females, methylation was found to be more frequent among Norwegian newborn girls (9%) and young adult women (8%) as compared to aging noncancer women in the US WHI study (5.5%; *p* < 0.001). While potential population- or ethnic-related differences may not be excluded, we observed no difference between participants belonging to different ethnic groups in the WHI study [68], and others [73] found *BRCA1* epimutations in WBC from newborn girls in a Middle-East population with a similar incidence to that recorded by us in Norwegian newborns. Moreover, in our previous study of *BRCA1* epimutations in ovarian cancer patients, we observed an age-related drop in methylation frequency among patients as well as controls [52]. While no significant difference in methylation related to age was observed within our recent WHI study [68], all women were > 50 years of age but only 14% were > 70 years at WBC sample collection, limiting the power to detect potential age differences. An age-related drop in methylation could be due to actual demethylation of a promoter, or it could be due to clonal shifts where *BRCA1*-unmethylated clones displace methylated ones. Thus markers of clonal shift like X-chromosome skewness as well as clonal hematopoiesis [74, 75] are known to increase in aging. Notably, in addition to the 20% of TNBC found to harbor constitutional *BRCA1* promoter methylation [31], we found another 10% of TNBC to harbor *BRCA1* epimutations in their tumor tissue but not WBC. While such *BRCA1* epimutations may have occurred in adult life, potentially restricted to the breast, it may also reflect an early (constitutional) event with a VEF in WBC below our detection limits or a selective loss of *BRCA1*-epimutated cells in the WBC population due to a clonal sweep in the bone marrow, but not affecting breast epithelium. Clearly, more data, preferably including longitudinal samples, as well as a comparison of *BRCA1* epimutations between different normal tissues in the same individuals, are needed to fully address whether *BRCA1* epimutations in some tissues like WBC are lost with aging.

While low-level mosaic constitutional epimutations are detected also in WBC in individuals harboring g*BRCA1* PV [52, 68], *BRCA1* mutations and epimutations seem to be mutually exclusive in breast cancer tissue [31, 33], indicating mutations and epimutations to be independent underlying risk factors.

Taken together, these findings strongly suggest normal tissue *BRCA1* epimutations to be primary constitutional epimutations, likely originating in a single cell at an early embryonic stage prior to germ-layer separation. *BRCA1* epimutations differ from epimutations in other tumor suppressors like *MLH1* and *MHS2* in several ways. Apart from the few families characterized by secondary constitutional *BRCA1* epimutations, contrasting *MLH1* and *MSH2* epimutations, primary constitutional *BRCA1* epimutations affect a relatively large part of the population, females in particular, and stands out as a significant contributor to TNBC and HGSOC development. While individuals harboring low-level mosaic *MLH1* epimutations are reported [76–78], most individuals harboring *MLH1* and all with secondary *MSH2* epimutations have a high VEF. As for epimutations with a high VEF approaching 0.5 in normal tissue, a pathogenic role in tumor tissue can be inferred from LOH or other marks of inactivation of the normal allele. In cases where a tumor arises from a cell with a *BRCA1* epimutation in an individual with a low level of *BRCA1* methylation, one would expect to see a clonal expansion into a tumor revealing a much higher VEF, in addition to LOH [31].

## 5. Future Directions

**Figure 6:**
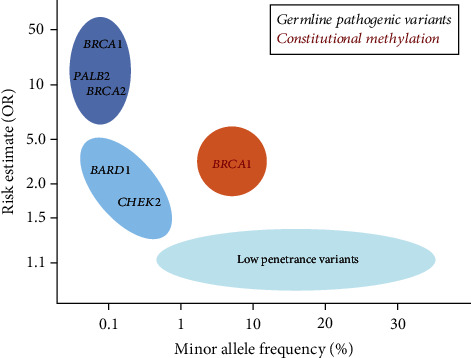
Population frequencies (*x*-axis) and associated risk estimates (OR; *y*-axis) for incident TNBC observed for constitutional *BRCA1* epimutations and selected high-, intermediate-, and low-risk germline genetic variants. Modified from [79].

Contemporary findings clearly indicate constitutional *BRCA1* epimutations to be a significant contributor to TNBC and HGSOC development, with a higher population frequency than genetic variants with similar HRs as well as a higher HR than genetic variants with similar population frequency (Figure 6). The findings, however, raise a number of questions to be addressed:

First, *how* do these primary constitutional *BRCA1* epimutations arise? While our data excludes dominant monogenetic inheritance [31], recent findings of mosaic *BRCA1* epimutations among patients from families with aggregation of breast/ovarian cancer not harboring germline variants [80] and a study reporting cases of *BRCA1* normal tissue epimutation covariance between mothers and daughters [73] open the possibility of multifactorial inheritance or more complex recombination events as well as potential roles of exogenous or endogenous factors in pregnancy [63]. Yet, for both studies, the number of individuals was small, and the findings need confirmation in independent studies. Considering the potential implications we now see for *BRCA1* epimutations, clearly, identifying individuals at particular risk for epimutations and/or identifying preventive measures are important targets for future studies.

Second, *when* do the *BRCA1* epimutations arise? The fact that they seem to affect tissue derived from all germ layers indicates an early origin, probably during the first weeks of gestation [81]. A clonal evolution of *BRCA1*-methylated ectodermal- and mesodermal-derived cancer cells from a common precursor is corroborated by the finding of *BRCA1* epimutations across normal tissues derived from all three germ layers as well as an allele-specific concordance of methylation in breast tumor, normal breast tissue, and WBC [31, 52]. The first weeks of gestation is a time involving several methylation/demethylation waves [82]. Pinpointing the exact timing of the epimutation may point toward specific embryonic events involved with the methylation and therefore may have huge implications for our understanding of early life events and human pathophysiology.

Third, how may the contribution of primary constitutional epimutations to cancer risk affect the *field of genetics*? At this stage, the HR for either HGSOC or TNBC found does not advocate predictive testing or genetic counselling at an individual level. Yet, the finding that *BRCA1* epimutations affect a substantial percentage of the population and are found to be associated with a large fraction of TNBC and HGSOC, may influence current modelling on cancer risk factors, with emphasis on the contribution of low-risk genes to cancer risk in the population. Considering *BRCA1* epimutations to arise as very early events, this may potentially affect epimutation concordance between monozygotic twins [81]. A key tool exploring overall genetic contribution to cancer risk is comparing cancer concordance in mono- versus dizygotic twins pairs [83]. In case there is a significant methylation concordance for *BRCA1* (and potentially other cancer risk genes as well) in monozygotic twins, current models for genetic contribution to disease risk will turn out to be overestimates and would have to be revised based on epigenetic knowledge.

Fourth, what is the impact on cancer *prevention*? The currently estimated HRs for TNBC and HGSOC may be a part of future stratification in personalized screening programs. However, perhaps more relevant in a short-term perspective; women diagnosed with spontaneous breast cancer are known to carry an increased risk for subsequent cancer of the ovary and secondary breast cancers [84, 85]. Based on the HR for TNBC and HGSOC related to *BRCA1* constitutional methylation [68], women diagnosed with these malignancies and found to harbor constitutional methylation may be considered at increased risk of a secondary tumor and could, therefore, be referred for special surveillance.

Fifth, are low-mosaic primary constitutional epimutations in other tumor suppressor genes risk factors for *other cancers*? This is a key question for the field in the immediate future. A rational approach would be to start by assessing genes that are epimutated in a certain fraction of a specific tumor type and reveal mosaic normal tissue epimutations at a population frequency high enough to enable testing.

Sixth, do primary constitutional epimutations in *BRCA1* (or other genes) affect the risk of *diseases other than cancer*? Given the role of mosaic *BRCA1* epimutations in TNBC and HGSOC, similar mechanisms may be the underlying causes of a multitude of diseases. Future studies on this are highly warranted.

## 6. Clinical Implications

The finding that constitutional primary *BRCA1* epimutations are an underlying cause of a substantial fraction of TNBC and HGSOC has significant implications for our understanding of the biology of tumorigenesis. Further research is needed to characterize the exact mechanism(s) by which such epimutations originate. Considering clinical implications, the HR for HGSOC and TNBC does not currently advocate routine testing for WBC *BRCA1* epimutations in healthy individuals. However, women diagnosed with breast cancer have an elevated risk of a secondary tumor [86], and it seems reasonable to test all TNBC and HGSOC for tissue methylation and offer WBC *BRCA1* methylation testing to women diagnosed with a *BRCA1* methylated TNBC or HGSOC tumor. Regarding cancer treatment, preliminary evidence indicates that *BRCA1* promoter methylation is associated with sensitivity to a PARP inhibitor in the primary setting in breast cancer [39] while results in metastatic disease are conflicting. More data linking tissue *BRCA1* methylation to therapy response as well as to other parameters, like RAD51 foci expression [87] and genomic signatures [10, 12] would be needed prior to any implementation of methylation testing for therapy prediction, outside clinical trials. Future studies should also assess the potential implications of *BRCA1* epimutations as predictive for PARP-inhibition in other tumor types, in which *BRCA1/2* mutations are observed (e.g., prostate cancer).

## Data Availability

Data sharing is not applicable to this article as no datasets were generated or analyzed during the current study.
